# Does APC/C^CDH1^ control the human brain size? An Editorial Highlight for https://doi.org/10.1111/jnc.14828

**DOI:** 10.1111/jnc.14835

**Published:** 2019-08-23

**Authors:** Maike F. Dohrn, Juan P. Bolaños

**Affiliations:** ^1^ Department of Neurology, Medical Faculty RWTH Aachen University Aachen Germany; ^2^ Institute of Functional Biology and Genomics CSIC, University of Salamanca Salamanca Spain; ^3^ Centro de Investigación Biomédica en Red sobre Fragilidad y Envejecimiento Saludable (CIBERFES) Institute of Biomedical Research of Salamanca Salamanca Spain

## Abstract

This editorial highlights a study by Rodriguez, Sanchez‐Moran et al. (2019) in the current issue of the *Journal of Neurochemistry*, in which the authors describe a microcephalic boy carrying the novel heterozygous *de novo* missense mutation c.560A> G; p.Asp187Gly in *Cdh1/Fzr1* encoding the APC/C E3‐ubiquitin ligase cofactor CDH1. A functional characterization of mutant APC/C^CDH1^ confirms an aberrant division of neural progenitor cells, a condition known to determine the mouse brain cortex size. These data suggest that APC/C^CDH1^ may contribute to the regulation of the human brain size.

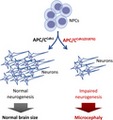

## Graphical abstract

This editorial highlights a study by Rodriguez, Sanchez‐Moran et al. (2019) in the current issue of the *Journal of Neurochemistry*, in which the authors describe a microcephalic boy carrying the novel heterozygous *de novo* missense mutation c.560A>G; p.Asp187Gly in *Cdh1/Fzr1* encoding the APC/C E3‐ubiquitin ligase cofactor CDH1. The mutation causes a substantial loss of anaphase‐promoting complex/cyclosome (APC/C) activity resulting in replicative stress of neural progenitor cells (NPCs) leading to impaired neurogenesis during the embryonic stage. Based on the patient’s phenotype, one can further hypothesize that this mutation leads to an antenatally reduced size of brain cortex (microcephaly).

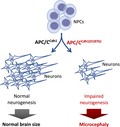

**Abbreviations used**
APC/C: anaphase‐promoting complex/cyclosome
CDH1/Fzr1: Cdc20‐homolog‐1/Fizzy‐related protein‐1
PFKFB3: 6‐phosphofructo‐2‐kinase/fructose‐2,6‐bisphosphatase‐3

Microcephaly is a brain development disorder resulting in a reduced size of both brain and head (Barkovich *et al.* 2012). Although the causes of microcephaly are diverse, many of them are genetic, a circumstance that has enabled to understand the physiology of cortical development (Thornton and Woods 2009). Some of these genes are involved in the control of mitotic function suggesting that the disease pathology is associated with aberrant neural progenitor cell proliferation. The anaphase‐promoting complex/cyclosome (APC/C) is an E3 ubiquitin ligase essential for the regulation of cell division (Garcia‐Higuera *et al.* 2008). Depending on the cell cycle stage, APC/C requires binding to either CDC20 or CDH1 (also known as Fzr1) for its full activity. To exit mitosis, APC/C needs to be activated by CDH1, and the APC/C^CDH1^ complex remains active during the G_1_ phase of the cell cycle as well as in G_0_ (postmitotic cells) (Garcia‐Higuera *et al.* 2008). As postmitotic cells, fully differentiated neurons show a highly active APC/C^CDH1^ complex, which continuously promotes the degradation of proteins such as 6‐phosphofructo‐2‐kinase/fructose‐2,6‐bisphosphatase‐3 (PFKFB3) (Herrero‐Mendez *et al.* 2009), ROCK2 (Bobo‐Jimenez *et al.* 2017), and cyclin B1 (Almeida *et al.* 2005) to regulate energy metabolism, dendritic integrity, memory, and survival (Bolaños 2016). Interestingly, during mouse brain development, APC/C^CDH1^ was found to control the division of neural progenitor cells to determine the correct size of the brain cortex (Delgado‐Esteban *et al.* 2013). Indeed, genetic deletion of *Cdh1* specifically during the embryonic stage impairs neurogenesis causing microcephaly (Delgado‐Esteban *et al.* 2013), thus posing the loss of APC/C^CDH1^ function as a potential cause of human microcephaly.

Our current knowledge on APC/C^CDH1^ function in the brain has been built on research performed in cellular and non‐human organismal models. Hence, the actual importance of this ubiquitin ligase in human brain pathophysiology has so far remained uncertain. In the current issue of the *Journal of Neurochemistry*, Rodríguez *et al. *(2019) report the first case of a putatively pathogenic, heterozygous missense mutation in *Cdh1* causing microcephaly, mental retardation, spasticity, and epilepsy in a 4‐year‐old boy. Assuming a functionally important role in brain development, the authors searched for potentially disease‐causing variants in the *Cdh1* gene screening several hundreds of whole exomes, all of which were previously sequenced in individuals with neurodevelopmental disorders of a potentially genetic origin. The heterozygous missense mutation c.560A>G; p.Asp187Gly was verified to have arisen *de novo* from healthy, non‐consanguineous parents of Spanish descend (Rodriguez *et al.* 2019). Besides severe antenatal microcephaly, the boy showed psychomotor retardation and refractory epilepsy, well‐known signs of microcephaly. To link the p.Asp187Gly mutation in *Cdh1* with the microcephaly phenotype, the authors functionally characterized the mutant protein. The finding that CDH1 protein abundance was substantially lower in the patient’s leucocytes when compared with those isolated from his parents (Rodriguez *et al.* 2019) strongly suggests an impaired E3 ubiquitin ligase APC/C activity. To confirm this, the authors engineered, by site‐directed mutagenesis, the mutant (c.560A>G) full‐length *Cdh1* cDNA. The implementation of mutant *Cdh1* in human HEK293T cells confirmed the reduced expression of CDH1 protein (Rodriguez *et al.* 2019). To ascertain whether APC/C activity was impaired, the authors determined the protein abundances of two well‐known substrates of APC/C^CDH1^, namely cyclin B1 (Almeida *et al.* 2005) and PFKFB3 (Herrero‐Mendez *et al.* 2009). Both protein abundances were found to be significantly increased in cells transfected with mutant *Cdh1* when compared with cells expressing wild‐type CDH1 (Rodriguez *et al.* 2019). Confocal imaging characterization of HEK293T cells expressing either the mutant or the wild‐type CDH1 protein confirmed exclusive nuclear localization of the mutant form (Rodriguez *et al.* 2019), a condition compatible with low CDH1 protein stability (Nagai *et al.* 2018). Moreover, analysis of the cell cycle distribution revealed a severe delay in the exit from mitosis in HEK293T cells expressing mutant CDH1 (Rodriguez *et al.* 2019). Thus, the functional characterization of human cells carrying mutant CDH1 confirms the typical features of APC/C^CDH1^ loss of function. Finally, the authors expressed either the mutant or the wild‐type CDH1 in mouse neural progenitor cells isolated from a *Cdh1*‐null genetic background. The analysis of cell cycle revealed an enlargement of the S‐phase in those cells expressing the mutant CDH1 (Rodriguez *et al.* 2019), that is, the typical feature of neurogenesis impairment causing microcephaly previously reported by the authors *via* genetic *Cdh1* ablation (Delgado‐Esteban *et al.* 2013). Altogether, these sets of elegant experiments strongly indicate that the novel missense mutation c.560A>G; p.Asp187Gly identified in human *Cdh1* results in APC/C loss of activity causing the typical neurogenesis impairment of microcephaly (Fig. 1).

**Figure 1:**
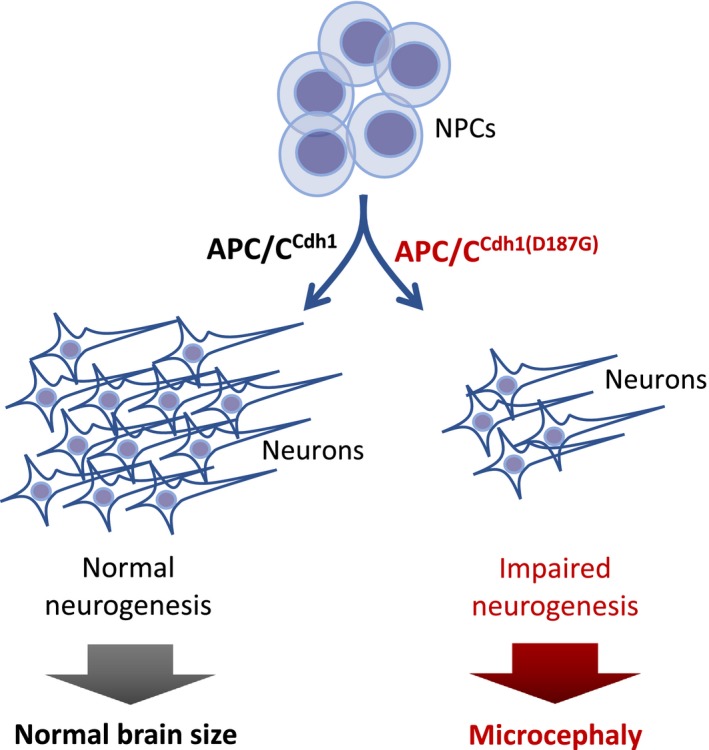
APC/C^CDH1(D187G)^ may cause microcephaly. The novel p.Asp187Gly (D187G) mutation found by Rodriguez, Sanchez‐Moran et al. (2019) in the human *Fzr1* gene causes a substantial loss of anaphase‐promoting complex/cyclosome (APC/C) activity. This results in replicative stress of neural progenitor cells (NPCs) leading to impaired neurogenesis (formation of new neurons) during the embryonic stage. Based on the patient’s phenotype, one can hypothesize that this mutation leads to an antenatally reduced size of the brain cortex (microcephaly).

It is intriguing, however, that this heterozygous *Cdh1* mutation is sufficient to develop such a severe phenotypic impact (Rodriguez *et al.* 2019). Primary microcephaly has already been associated with disturbed mitosis, but the underlying mode of inheritance has typically been autosomal recessive (Thornton and Woods, 2009; Faheem *et al.,* 2015). Furthermore, a dominant negative effect is usually not expected in a loss of function pathomechanism. The authors explain that this observation might be ascribed to the low intolerance score of the *Cdh1* gene (Firth *et al.* 2009; Lek *et al.,* 2016) indicating that the loss of function of one mutated allele cannot be compensated by the wild type (Rodriguez *et al.* 2019). While this may be a suitable explanation, it should be noticed that heterozygous Cdh1‐knockout mice (*Cdh1^+/‐^*) show no alterations in the cell division of neural progenitors, which are identical to that found in the wild‐type animals (Delgado‐Esteban *et al.* 2013). These data suggest that the loss of mutant p.Asp187Gly CDH1 protein may not be sufficient to explain the lack of APC/C function. One methodological limitation of this work might be that additional, regulatory variants placed within an intronic region of *Cdh1* could not be excluded by whole exome sequencing. Whether there is another functionally relevant splice mutation lingering on the trans allele in this patient remains to be elucidated. Co‐segregation analyses, which are in principle an important step to approach for a variant of unknown significance, were not eligible in this case, as the parents were excluded to be carriers due to the *in trio* sequencing; thus, it was not necessary to gather further information on siblings.

In conclusion, the study by Rodriguez, Sanchez‐Moran *et al. *(2019) demonstrates in an excellent way, how a precise interplay of clinical phenotyping, modern sequencing techniques, and functional models can help to identify the molecular genetic cause of even a very rare condition and to understand the underlying molecular mechanisms leading to brain development disorders. Whether the mutant p.Asp187Gly CDH1 protein has any structural feature that impairs its ability to interact with APC/C substrates is another issue remaining to be explored. To elucidate this question and to unambiguously demonstrate the cause–effect relationship between the p.Asp187Gly *Cdh1* mutation and microcephaly, it would be needed to characterize a knock‐in mouse genetically engineered to harbor such a mutation in heterozygosity. Addressing this aspect would shed light not only onto the molecular mechanism of CDH1 protein–protein interaction in APC/C function but also onto the regulation of human cortical brain size and, hence, intellectual ability.
